# Current Awareness on Comparative and Functional Genomics

**DOI:** 10.1002/cfg.356

**Published:** 2004


**Comparative and Functional Genomics**
*Comp Funct Genom* 2004; **5**: 563–570
Published online in Wiley InterScience (www.interscience.wiley.com). **DOI:** 10.1002cfg.356

### Current awareness on comparative and functional genomics

CURRENT AWARENESS - USE THE LINKS ONLINE

### 1 Reviews & symposia

2004. Special issue - Expression Profiling within the Central Nervous System - Part II. *Neurochem Res* **29:** (6)

Abbott CA. 2003. 11th Intelligent Systems for Molecular Biology 2003 (ISMB 2003) (Conference Review). *Comp Funct Genom* **4:** (6) 654.

Aboobaker AA, Blaxter ML. 2004. Functional genomics for parasitic nematodes and platyhelminths (Review). *Trends Parasitol* **20:** (4) 178.

Allaby RG, Woodwark M. 2004. Phylogenetics in the bioinformatics culture of understanding (Review). *Comp Funct Genom* **5:** (2) 128.

Amoutzias GD, Robertson DL, Bornberg-Bauer E. 2004. The evolution of protein interaction networks in regulatory proteins (Conference Review). *Comp Funct Genom* **5:** (1) 79.

Atreas ND, Karanikas C, Polychronidou P. 2004. Signal analysis on strings for immune-type pattern recognition (Conference Review). *Comp Funct Genom* **5:** (1) 69.

Beynon RJ. 2004. Enabling proteomics: The need for an extendable 'workbench' for user-configurable solutions (Conference Review). *Comp Funct Genom* **5:** (1) 52.

Blaschke C, Yeh A, Hirschman L, Valencia A. 2003. ISMB 2003 Text Mining SIG Meeting report (Conference Review). *Comp Funct Genom* **4:** (6) 667.

Blaschke C, Hirschman L, Yeh A, Valencia A. 2003. Critical assessment of information extraction systems in biology (Conference Review). *Comp Funct Genom* **4:** (6) 674.

Borkovich KA, Alex LA, Yarden O, Freitag M, Turner GE, Read ND, Seiler S, Bell-Pedersen D, Paietta J, Plesofsky N *et al*. 2004. Lessons from the genome sequence of *Neurospora crassa*: Tracing the path from genomic blueprint to multicellular organism. *Microbiol Mol Biol Rev* **68:** (1) 1.

Butterfield DA. 2004. Proteomics: A new approach to investigate oxidative stress in Alzheimer's disease brain (Review). *Brain Res* **1000:** (1-2) 1.

Cambon-Thomsen A, Ducournau P, Gourraud P, Pontille D. 2003. Biobanks for genomics and genomics for biobanks (Conference Review). *Comp Funct Genom* **4:** (6) 628.

Carbonaro M. 2004. Proteomics: Present and future in food quality evaluation. *Trends Food Sci Technol* **15:** (3-4) 209.

Domon B, Broder S. 2004. Implications of new proteomics strategies for biology and medicine. *J Proteome Res* **3:** (2) 253.

Dorsett Y, Tuschl T. 2004. siRNAs: Applications in functional genomics and potential as therapeutics. *Nat Rev Drug Discov* **3:** (4) 318.

Dunham I, Beare DM, Collins JE. 2003. The characteristics of human genes: Analysis of human chromosome 22 (Conference Review). *Comp Funct Genom* **4:** (6) 635.

ffrench-Constant RH, Daborn PJ, Le Goff G. 2004. The genetics and genomics of insecticide resistance (Review). *Trends Genet* **20:** (3) 163.

Forejt J, Vacik T, Gregorova S. 2003. Segmental trisomy of mouse chromosome 17: Introducing an alternative model of Down's syndrome (Conference Review). *Comp Funct Genom* **4:** (6) 647.

Gabaldon T, Huynen MA. 2004. Prediction of protein function and pathways in the genome era. *Cell Mol Life Sci* **61:** (7-8) 930.

Gheorghe M, Mitrana V. 2004. A formal language-based approach in biology (Conference Review). *Comp Funct Genom* **5:** (1) 91.

Giavitto JL, Malcolm G, Michel O. 2004. Rewriting systems and the modelling of biological systems (Conference Review). *Comp Funct Genom* **5:** (1) 95.

Hanash S. 2004. Building a foundation for the human proteome: The role of the human proteome organization. *J Proteome Res* **3:** (2) 197.

Hemelaar J, Galardy PJ, Borodovsky A, Kessler BA, Ploegh HL, Ovaa H. 2004. Chemistry-based functional proteomics: Mechanism-based activity-profiling tools for ubiquitin and ubiquitin-like specific proteases. *J Proteome Res* **3:** (2) 268.

Koonin EV. 2003. Comparative genomics, minimal gene-sets and the last universal common ancestor. *Nat Rev Microbiol* **1:** (2) 127.

Kramer JA, Pettit SD, Amin RP, Bertram TA, Car B, Cunningham M, Curtiss SW, Davis JW, Kind C, Lawton M *et al*. 2004. Overview of the application of transcription profiling using selected nephrotoxicants for toxicology assessment. *Environ Health Perspect* **112:** (4) 460.

Lindon JC, Holmes E, Nicholson JK. 2004. Metabonomics and its role in drug development and disease diagnosis. *Expert Rev Mol Diagn* **4:** (2) 189.

Lord P, Stevens R. 2003. ISMB 2003 Bio-ontologies SIG and Sixth Annual Bio-ontologies Meeting report (Conference Review). *Comp Funct Genom* **4:** (6) 663.

Marko-Varga G, Fehniger TE. 2004. Proteomics and disease - The challenges for technology and discovery. *J Proteome Res* **3:** (2) 167.

Martienssen RA, Rabinowicz PD, O'Shaughnessy A, McCombie WR. 2004. Sequencing the maize genome. *Curr Opin Plant Biol* **7:** (2) 102.

Miyagishi M, Matsumoto S, Taira K. 2004. Generation of an shRNAi expression library against the whole human transcripts. *Virus Res* **102:** (1) 117.

Patel M, Nagl S. 2004. MicroCore: Mapping genome expression to cell

In order to keep subscribers up-to-date with the latest developments in their field, this current awareness service is provided by John Wiley & Sons and contains newly-published material on comparative and functional genomics. Each bibliography is divided into 16 sections. 1 Reviews & symposia; 2 General; 3 Large-scale sequencing and mapping; 4 Evolutionary genomics; 5 Comparative genomics; 6 Pathways, gene families and regulons; 7 Pharmacogenomics; 8 EST, cDNA and other clone resources; 9 Functional genomics; 10 Transcriptomics; 11 Proteomics; 12 Protein structural genomics; 13 Metabolomics; 14 Genomic approaches to development; 15 Technological advances; 16 Bioinformatics. Within each section, articles are listed in alphabetical order with respect to author. If, in the preceding period, no publications are located relevant to any one of these headings, that section will be omitted.

Copyright © 2004 John Wiley & Sons, Ltd.

pathways and networks (Conference Review). *Comp Funct Genom* **5:** (1) 75.
Paton RC, Toh CH. 2004. Computational aspects of protein functionality (Conference Review). *Comp Funct Genom* **5:** (1) 85.
Pennie W, Pettit SD, Lord PG. 2004. Toxicogenomics in risk assessment: An overview of an HESI collaborative research program. *Environ Health Perspect* **112:** (4) 417.
Petricoin E, Wulfkuhle J, Espina V, Liotta LA. 2004. Clinical proteomics: Revolutionizing disease detection and patient tailoring therapy. *J Proteome Res* **3:** (2) 209.
Pettifer SR, Sinnott JR, Attwood TK. 2004. UTOPIA - User-friendly tools for operating informatics applications (Conference Review). *Comp Funct Genom* **5:** (1) 56.
Ravichandran LV, Dean NM, Marcusson EG. 2004. Use of antisense oligonucleotides in functional genomics and target validation (Review). *Oligonucleotides* **14:** (1) 49.
Reddy KS, Perrotta PL. 2004. Proteomics in transfusion medicine (Review). *Transfusion* **44:** (4) 601.
Rees-Unwin KS, Morgan GJ, Davies FE. 2004. Proteomics and the haematologist (Review). *Clin Lab Haematol* **26:** (2) 77.
Schachter V. 2003. ISMB 2003 BioPathways SIG and 5th BioPathways Meeting (Conference Review). *Comp Funct Genom* **4:** (6) 660.
Sepper R, Prikk K. 2004. Proteomics: Is it an approach to understand the progression of chronic lung disorders? *J Proteome Res* **3:** (2) 277.
Smith RD, Shen YF, Tang KQ. 2004. Ultrasensitive and quantitative analyses from combined separations-mass spectrometry for the characterization of proteomes. *Account Chem Res* **37:** (4) 269.
Sugimoto A. 2004. High-throughput RNAi in *Caenorhabditis elegans*: Genome-wide screens and functional genomics. *Differentiation* **72:** (2-3) 81.
Sun W, He FC. 2004. Application of proteomics in hepatic diseases research. *Sci China C Life Sci* **47:** (2) 101.
Tosato V, Bruschi CV. 2004. Knowledge of the *Bacillus subtilis* genome: Impacts on fundamental science and biotechnology (Review). *Appl Microbiol Biotechnol* **64:** (1) 1.
Ulrich R, Rockett J, Gibson G, Pettit S. 2004. Overview of an interlaboratory collaboration on evaluating the effects of model hepatotoxicants on hepatic gene expression. *Environ Health Perspect* **112:** (4) 423.
Vasquez GB, Zullo SJ, Barker PE. 2004. Standards requirements for systems biology approaches to health care: Mitochondrial proteomics (Review). *Mitochondrion* **3:** (4) 205.
Vlachos C, Gregory R, Paton RC, Saunders JR, Wu QH. 2004. Individual-based modelling of bacterial ecologies and evolution (Conference Review). *Comp Funct Genom* **5:** (1) 100.
Weston AD, Hood L. 2004. Systems biology, proteomics, and the future of health care: Toward predictive, preventative, and personalized medicine. *J Proteome Res* **3:** (2) 179.
Zerkowski HR, Grussenmeyer T, Matt P, Grapow M, Engelhardt S, Lefkovits I. 2004. Proteomics strategies in cardiovascular research. *J Proteome Res* **3:** (2) 200.

### 3 Large-scale sequencing and mapping

Abrahamsen M, Templeton T, Enomoto S, Abrahante J, Zhu G, Lancto C, Deng M, Liu C, Widmer G, Tzipori S *et al*. 2004. Complete genome sequence of the apicomplexan, *Cryptosporidium parvum*. *Science* **304:** (5669) 441.
Bruggemann H, Gottschalk G. 2004. Insights in metabolism and toxin production from the complete genome sequence of *Clostridium tetani*. *Anaerobe* **10:** (2) 53.
Chen R, Sodergren E, Weinstock GM, Gibbs RA. 2004. Dynamic building of a BAC clone tiling path for the Rat Genome Sequencing Project. *Genome Res* **14:** (4) 679.
Dietrich FS, Voegeli S, Brachat S, Lerch A, Gates K, Steiner S, Mohr C, Pohlmann R, Luedi P, Choi SD, Wing RA, Flavier A, Gaffney TD, Philippsen P. 2004. The *Ashbya gossypii* genome as a tool for mapping the ancient *Saccharomyces cerevisiae* genome. *Science* **304:** (5668) 304.
Dobbins AT, George M, Basham DA, Ford ME, Houtz JM, Pedulla ML, Lawrence JG, Hatfull GF, Hendrix RW. 2004. Complete genomic sequence of the virulent *Salmonella* bacteriophage SP6. *J Bacteriol* **186:** (7) 1933.
Dreyer H, Steiner G. 2004. The complete sequence and gene organization of the mitochondrial genome of the gadilid scaphopod *Siphonondentalium lobatum* (Mollusca). *Mol Phylogenet Evol* **31:** (2) 605.
Gardiner J, Schroeder S, Polacco ML, Sanchez-Villeda H, Fang ZW, Morgante M, Landewe T, Fengler K, Useche F, Hanafey M *et al*. 2004. Anchoring 9,371 maize expressed sequence tagged unigenes to the bacterial artificial chromosome contig map by two-dimensional overgo hybridization. *Plant Physiol* **134:** (4) 1317.
Krzywinski M, Wallis J, Gosele C, Bosdet I, Chiu R, Graves T, Hummel O, Layman D, Mathewson C, Wye N *et al*. 2004. Integrated and sequence-ordered BAC and YAC-based physical maps for the rat genome. *Genome Res* **14:** (4) 766.
Kwitek AE, Gullings-Handley J, Yu JM, Carlos DC, Orlebeke K, Nie J, Eckert J, Lemke A, Andrae JW, Bromberg S *et al*. 2004. High-density rat radiation hybrid maps containing over 24,000 SSLPs, genes, and ESTs provide a direct link to the rat genome sequence. *Genome Res* **14:** (4) 750.
Lapuk A, Volik S, Vincent R, Chin K, Kuo WL, De Jong P, Collins C, Gray JW. 2004. Computational BAC clone contig assembly for comprehensive genome analysis. *Gene Chromosomes Cancer* **40:** (1) 66.
Makova KD, Yang S, Chiaromonte F. 2004. Insertions and deletions are male biased too: A whole-genome analysis in rodents. *Genome Res* **14:** (4) 567.
Malek JA, Wierzbowski JM, Tao W, Bosak SA, Saranga DJ, Doucette-Stamm L, Smith DR, McEwan PJ, McKernan KJ. 2004. Protein interaction mapping on a functional shotgun sequence of *Rickettsia sibirica*. *Nucleic Acids Res* **32:** (3) 1059.
Matsuzaki M, Misumi O, Shin-I T, Maruyama S, Takahara M, Miyagishima SY, Mori T, Nishida K, Yagisawa F, Nishida K *et al*. 2004. Genome sequence of the ultrasmall unicellular red alga *Cyanidioschyzon merolae* 10D. *Nature* **428:** (6983) 653.
Meurice G, Jacob D, Deborde C, Chaillou S, Rouault A, Leverrier P, Jan G, Thierry A, Maillard MB, Amet P, Lalande M, Zagorec M, Boyaval P, Dimova D. 2004. Whole genome sequencing project of a dairy *Propionibacterium freudenreichii* subsp *shermanii* genome: Progress and first bioinformatic analysis. *Lait* **84:** (1-2) 15.
Venter JC, Remington K, Heidelberg JF, Halpern AL, Rusch D, Eisen JA, Wu DY, Paulsen I, Nelson KE, Nelson W *et al*. 2004. Environmental genome shotgun sequencing of the Sargasso Sea. *Science* **304:** (5667) 66.
Wilder SP, Bihoreau MT, Argoud K, Watanabe TK, Lathrop M, Gauguier D. 2004. Integration of the rat recombination and EST maps in the rat genomic sequence and comparative mapping analysis with the mouse genome. *Genome Res* **14:** (4) 758.

### 4 Evolutionary genomics

Bakker FT, Culham A, Hettiarachi P, Touloumenidou T, Gibby M. 2004. Phylogeny of *Pelargonium* (Geraniaceae) based on DNA sequences from three genomes. *Taxon* **53:** (1) 17.
Bourque G, Pevzner PA, Tesler G. 2004. Reconstructing the genomic architecture of ancestral mammals: Lessons from human, mouse, and rat genomes. *Genome Res* **14:** (4) 507.
Caimi K, Cataldi A. 2004. A fragment of 21 ORFs around the direct repeat (DR) region of *Mycobacterium tuberculosis* is absent from the other sequenced mycobacterial genomes: Implications for the evolution of the DR region. *Comp Funct Genom* **5:** (2) 116.
Campanella JJ, Larko D, Smalley J. 2003. A molecular phylogenomic analysis of the ILRI-like family of IAA amidohydrolase genes. *Comp Funct Genom* **4:** (6) 584.
Cooper GM, Brudno M, Stone EA, Dubchak I, Batzoglou S, Sidow A. 2004. Characterization of evolutionary rates and constraints in three mammalian genomes. *Genome Res* **14:** (4) 539.
Counterman BA, Ortiz Barrientos D, Noor MAF. 2004. Using comparative genomic data to test for fast-x evolution. *Evolution* **58:** (3) 656.
Ghedin E, Bringaud F, Peterson J, Myler P, Berriman M, Ivens A, Andersson B, Bontempi E, Eisen J, Angiuoli S *et al*. 2004. Gene synteny and evolution of genome architecture in trypanosomatids. *Mol Biochem Parasitol* **134:** (2) 183.

Copyright © 2004 John Wiley & Sons, Ltd. 

Gibbs RA, Weinstock GM, Metzker ML, Muzny DM, Sodergren EJ, Scherer S, Scott G, Steffen D, Worley KC, Burch PE *et al*. 2004. Genome sequence of the Brown Norway rat yields insights into mammalian evolution. *Nature* **428:** (6982) 493.

Kellis M, Birren BW, Lander ES. 2004. Proof and evolutionary analysis of ancient genome duplication in the yeast *Saccharomyces cerevisiae*. *Nature* **428:** (6983) 617.

Sarkar SF, Guttman DS. 2004. Evolution of the core genome of *Pseudomonas syringae*, a highly clonal, endemic plant pathogen. *Appl Environ Microbiol* **70:** (4) 1999.

Shenoy AR, Sivakumar K, Krupa A, Srinivasan N, Visweswariah SS. 2004. A survey of nucleotide cyclases in Actinobacteria: Unique domain organization and expansion of the class III cyclase family in *Mycobacterium tuberculosis*. *Comp Funct Genom* **5:** (1) 17.

Stefancsik R, Randall JD, Mao C, Sarkar S. 2003. Structure and sequence of the human fast skeletal troponin T (TNNT3) gene: Insight into the evolution of the gene and the origin of the developmentally regulated isoforms. *Comp Funct Genom* **4:** (6) 609.

Taylor MS, Ponting CP, Copley RR. 2004. Occurrence and consequences of coding sequence insertions and deletions in mammalian genomes. *Genome Res* **14:** (4) 555.

Tsolaki A, Hirsh A, DeRiemer K, Enciso J, Wong M, Hannan M, De la Salmoniere Y, Aman K, Kato-Maeda M, Small P. 2004. Functional and evolutionary genomics of *Mycobacterium tuberculosis*: Insights from genomic deletions in 100 strains. *Proc Natl Acad Sci U S A* **101:** (14) 4865.

Yap VB, Pachter L. 2004. Identification of evolutionary hotspots in the rodent genomes. *Genome Res* **14:** (4) 574.

Zhang ZD, Burch PE, Cooney AJ, Lanz RB, Pereira FA, Wu JQ, Gibbs RA, Weinstock G, Wheeler DA. 2004. Genomic analysis of the nuclear receptor family: New insights into structure, regulation, and evolution from the rat genome. *Genome Res* **14:** (4) 580.

### 5 Comparative genomics

Brudno M, Poliakov A, Salamov A, Cooper G, Sidow A, Rubin E, Solovyev V, Batzoglou S, Dubchak I. 2004. Automated whole-genome multiple alignment of rat, mouse, and human. *Genome Res* **14:** (4) 685.

Jensen-Seaman M, Furey T, Payseur B, Lu Y, Roskin K, Chen C, Thomas M, Haussler D, Jacob H. 2004. Comparative recombination rates in the rat, mouse, and human genomes. *Genome Res* **14:** (4) 528.

Kolbe D, Taylor J, Elnitski L, Eswara P, Li J, Miller W, Hardison R, Chiaromonte F. 2004. Regulatory potential scores from genome wide three-way alignments of human, mouse and rat. *Genome Res* **14:** (4) 700.

Kwon JY, Hong M, Choi MS, Kang SJ, Duke K, Kim S, Lee SH, Lee JH. 2004. Ethanol-response genes and their regulation analyzed by a microarray and comparative genomic approach in the nematode *Caenorhabditis elegans*. *Genomics* **83:** (4) 600.

Nascimento A, Ko A, Martins E, Monteiro-Vitorello C, Ho P, Haake D, Verjovski-Almeida S, Hartskeerl R, Marques M, Oliveira M *et al*. 2004. Comparative genomics of two *Leptospira interrogans* serovars reveals novel insights into physiology and pathogenesis. *J Bacteriol* **186:** (7) 2164.

Plotkin JB, Dushoff J, Fraser HB. 2004. Detecting selection using a single genome sequence of *M. tuberculosis* and *P. falciparum*. *Nature* **428:** (6986) 942.

Seshadri G, Myers GSA, Tettelin H, Eisen JA, Heidelberg JF, Dodson RJ, Davidsen TM, DeBoy RT, Fouts DE, Haft DH *et al*. 2004. Comparison of the genome of the oral pathogen *Treponema denticola* with other spirochete genomes. *Proc Natl Acad Sci U S A* **101:** (15) 5646.

Severson DW, DeBruyn B, Lovin DD, Brown SE, Knudson DL, Morlais I. 2004. Comparative genome analysis of the yellow fever mosquito *Aedes aegypti* with *Drosophila melanogaster* and the malaria vector mosquito *Anopheles gambiae*. *J Hered* **95:** (2) 103.

Twigger SN, Nie J, Ruotti V, Yu JM, Chen D, Li DW, Mathis J, Narayanasamy V, Gopinath GR, Pasko D *et al*. 2004. Integrative genomics: *In silico* coupling of rat physiology and complex traits with mouse and human data. *Genome Res* **14:** (4) 651.

Ventura M, Canchaya C, Pridmore RD, Brussow H. 2004. The prophages of *Lactobacillus johnsonii* NCC 533: Comparative genomics and transcription analysis. *Virology* **320:** (2) 229.

Yang S, Smit AF, Schwartz S, Chiaromonte F, Roskin KM, Haussler D, Miller W, Hardison RC. 2004. Patterns of insertions and their covariation with substitutions in the rat, mouse, and human genomes. *Genome Res* **14:** (4) 517.

### 7 Pharmacogenomics

Ai JK, Zhang ZW, Xin DQ, Zhu HJ, Yan QJ, Xin ZC, Na YQ, Guo YL. 2004. Identification of over-expressed genes in human renal cell carcinoma by combining suppression subtractive hybridization and cDNA library array. *Sci China C Life Sci* **47:** (2) 148.

Amann K, Ridinger H, Rutenberg C, Ritz E, Mall G, Maercker C. 2003. Gene expression profiling on global cDNA arrays gives hints concerning potential signal transduction pathways involved in cardiac fibrosis of renal failure. *Comp Funct Genom* **4:** (6) 571.

Amin RA, Vickers AE, Sistare F, Thompson KL, Roman RJ, Lawton M, Kramer J, Hamadeh HK, Collins J, Grissom S *et al*. 2004. Identification of putative gene-based markers of renal toxicity. *Environ Health Perspect* **112:** (4) 465.

Arai M, Yokosuka O, Fukai K, Imazeki F, Chiba T, Sumi H, Kato M, Takiguchi M, Saisho H, Muramatsu M *et al*. 2004. Gene expression profiles in liver regeneration with oval cell induction. *Biochem Biophys Res Commun* **317:** (2) 370.

Baetz K, McHardy L, Gable K, Tarling T, Reberioux D, Bryan J, Andersen RJ, Dunn T, Hieter P, Roberge M. 2004. Yeast genome-wide drug-induced haploinsufficiency screen to determine drug mode of action. *Proc Natl Acad Sci U S A* **101:** (13) 4525.

Baker VA, Harries HM, Waring JF, Duggan CM, Ni HA, Jolly RA, Yoon LW, De Souza AT, Schmid JE, Brown RH *et al*. 2004. Clofibrate-induced gene expression changes in rat liver: A cross-laboratory analysis using membrane cDNA arrays. *Environ Health Perspect* **112:** (4) 428.

Borozdenkova S, Westbrook JA, Patel V, Wait R, Bolad I, Burke MM, Bell AD, Banner NR, Dunn MJ, Rose ML. 2004. Use of proteomics to discover novel markers of cardiac allograft rejection. *J Proteome Res* **3:** (2) 282.

Bullinger L, Dohner K, Bair E, Frohling S, Schlenk RF, Tibshirani R, Dohner H, Pollack JR. 2004. Use of gene-expression profiling to identify prognostic subclasses in adult acute myeloid leukemia. *N Engl J Med* **350:** (16) 1605.

Butura A, Johansson I, Nilsson K, Warngard L, Ingelman-Sundberg M, Schuppe-Koistinen I. 2004. Differentiation of human hepatoma cells during confluence as revealed by gene expression profiling. *Biochem Pharmacol* **67:** (7) 1249.

Chaurand P, Schwartz S, Caprioli R. 2004. Assessing protein patterns in disease using imaging mass spectrometry. *J Proteome Res* **3:** (2) 245.

Chertov O, Biragyn A, Kwak LW, Simpson JT, Boronina T, Hoang VM, Prieto DA, Conrads TP, Veenstra TD, Fisher RJ. 2004. Organic solvent extraction of proteins and peptides from serum as an effective sample preparation for detection and identification of biomarkers by mass spectrometry. *Proteomics* **4:** (4) 1195.

Chu TM, Deng SB, Wolfinger R, Paules RS, Hamadeh HK. 2004. Cross-site comparison of gene expression data reveals high similarity. *Environ Health Perspect* **112:** (4) 449.

Coen M, Ruepp SU, Lindon JC, Nicholson JK, Pognan F, Lenz EM, Wilson ID. 2004. Integrated application of transcriptomics and metabonomics yields new insight into the toxicity due to paracetamol in the mouse. *J Pharmaceut Biomed Anal* **35:** (1) 93.

Coulouarn C, Lefebvre G, Derambure C, Lequerre T, Scotte M, Francois A, Cellier D, Daveau M, Salier JP. 2004. Altered gene expression in acute systemic inflammation detected by complete coverage of the human liver transcriptome. *Hepatology* **39:** (2) 353.

Cromer A, Carles A, Millon R, Ganguli G, Chalmel F, Lemaire F, Young J, Dembele D, Thibault C, Muller D *et al*. 2004. Identification of genes associated with tumorigenesis and metastatic potential of hypopharyngeal cancer by microarray analysis. *Oncogene* **23:** (14) 2484.

Dangond F, Hwang D, Camelo S, Pasinelli P, Frosch MP, Stephanopoulos G, Stephanopoulos G, Brown RH, Gullans SR. 2004. Molecular signature of late-stage human ALS revealed by expression profiling of postmortem spinal cord gray matter. *Physiol Genomics* **16:** (2) 229.

Copyright © 2004 John Wiley & Sons, Ltd. 

Dhodda VK, Sailor KA, Bowen KK, Vemuganti R. 2004. Putative endogenous mediators of preconditioning-induced ischemic tolerance in rat brain identified by genomic and proteomic analysis. *J Neurochem* **89:** (1) 73.

Ding SJ, Li Y, Shao XX, Zhou H, Zeng R, Tang ZY, Xia QC. 2004. Proteome analysis of hepatocellular carcinoma cell strains, MHCC97-H and MHCC97-L, with different metastasis potentials. *Proteomics* **4:** (4) 982.

Fehniger TE, Sato-Folatre JG, Malmstrom J, Berglund M, Lindberg C, Brange C, Lindberg H, Marko-Varga G. 2004. Exploring the context of the lung proteome within the airway mucosa following allergen challenge. *J Proteome Res* **3:** (2) 307.

Fernandez-Arenas E, Molero G, Nombela C, Diez-Orejas R, Gil C. 2004. Contribution of the antibodies response induced by a low virulent *Candida albicans* strain in protection against systemic candidiasis. *Proteomics* **4:** (4) 1204.

Fernandez-Teijeiro A, Betensky RA, Sturla LM, Kim JYH, Tamayo P, Pomeroy SL. 2004. Combining gene expression profiles and clinical parameters for risk stratification in medulloblastomas. *J Clin Oncol* **22:** (6) 994.

Gatti L, Beretta GL, Carenini N, Corna E, Zunino F, Perego P. 2004. Gene expression profiles in the cellular response to a multinuclear platinum complex. *Cell Mol Life Sci* **61:** (7-8) 973.

Glinsky GV, Higashiyama T, Glinskii AB. 2004. Classification of human breast cancer using gene expression profiling as a component of the survival predictor algorithm. *Clin Cancer Res* **10:** (7) 2272.

Hayman MW, Przyborski SA. 2004. Proteomic identification of biomarkers expressed by human pluripotent stem cells. *Biochem Biophys Res Commun* **316:** (3) 918.

Hui LJ, Zhang X, Wu X, Lin ZX, Wang QK, Li YX, Hu GX. 2004. Identification of alternatively spliced mRNA variants related to cancers by genome-wide ESTs alignment. *Oncogene* **23:** (17) 3013.

Ikehara M, Oshita F, Sekiyama A, Hamanaka N, Saito H, Yamada K, Noda K, Kameda Y, Miyagi Y. 2004. Genome-wide cDNA microarray screening to correlate gene expression profile with survival in patients with advanced lung cancer. *Oncol Rep* **11:** (5) 1041.

Ishibashi K, Miura NN, Adachi Y, Ogura N, Tamura H, Tanaka S, Ohno N. 2004. DNA array analysis of altered gene expression in human leukocytes stimulated with soluble and particulate forms of *Candida* cell wall β-glucan. *Int J Immunopharmacol* **4:** (3) 387.

Iwamoto K, Kakiuchi C, Bundo M, Ikeda K, Kato T. 2004. Molecular characterization of bipolar disorder by comparing gene expression profiles of postmortem brains of major mental disorders. *Mol Psychiatry* **9:** (4) 406.

Johnson CD, Balagurunathan Y, Tadesse MG, Falahatpisheh MH, Brun M, Walker MK, Dougherty ER, Ramos KS. 2004. Unraveling gene-gene interactions regulated by ligands of the aryl hydrocarbon receptor. *Environ Health Perspect* **112:** (4) 403.

Johnson JR, Florens L, Carucci DJ, Yates JR. 2004. Proteomics in malaria. *J Proteome Res* **3:** (2) 296.

Juuti-Uusitalo K, Maki M, Kaukinen K, Collin P, Visakorpi T, Vihinen M, Kainulainen H. 2004. cDNA microarray analysis of gene expression in coeliac disease jejunal biopsy samples. *J Autoimmun* **22:** (3) 249.

Kamino H, Hiratsuka M, Toda T, Nishigaki R, Osaki M, Ito H, Inoue T, Oshimura M. 2003. Searching for genes involved in arteriosclerosis: Proteomic analysis of cultured human umbilical vein endothelial cells undergoing replicative senescence. *Cell Struct Funct* **28:** (6) 495.

Kiyosawa H, Kawashima T, Silva D, Petrovsky N, Hasegawa Y, Sakai K, Hayashizaki Y. 2004. Systematic genome-wide approach to positional candidate cloning for identification of novel human disease genes. *Intern Med* **34:** (3) 79.

Kuhn E, Wu J, Karl J, Liao H, Zolg W, Guild B. 2004. Quantification of C-reactive protein in the serum of patients with rheumatoid arthritis using multiple reaction monitoring mass spectrometry and $^{13}C$-labeled peptide standards. *Proteomics* **4:** (4) 1175.

Lee BC, Cha K, Avraham S, Avraham HK. 2004. Microarray analysis of differentially expressed genes associated with human ovarian cancer. *Int J Oncol* **24:** (4) 847.

Lein ES, Zhao XY, Gage FH. 2004. Defining a molecular atlas of the hippocampus using DNA microarrays and high-throughput *in situ* hybridization. *J Neurosci* **24:** (15) 3879.

Li JN, White N, Zhang Z, Rosenzweig J, Mangold LA, Partin AW, Chan DW. 2004. Detection of prostate cancer using serum proteomics pattern in a histologically confirmed population. *J Urol* **171:** (5) 1782.

Li KC, Yuan S. 2004. A functional genomic study on NCI's anticancer drug screen. *Pharmacogenomics J* **4:** (2) 127.

Lomnytska M, Lukiyanchuk V, Hellman U, Soucheinytskyi S. 2004. Transforming growth factor-β1-regulated proteins in human endothelial cells identified by two-dimensional gel electrophoresis and mass spectrometry. *Proteomics* **4:** (4) 995.

Mazzanti C, Zeiger MA, Costourous N, Umbricht C, Westra WH, Smith D, Somervell H, Bevilacqua G, Alexander HR, Libutti SK. 2004. Using gene expression profiling to differentiate benign *versus* malignant thyroid tumors. *Cancer Res* **64:** (8) 2898.

Meehan KL, Sadar MD. 2004. Quantitative profiling of LNCaP prostate cancer cells using isotope-coded affinity tags and mass spectrometry. *Proteomics* **4:** (4) 1116.

Melle C, Kaufmann R, Hommann M, Bleul A, Driesch D, Ernst G, Von Eggeling F. 2004. Proteomic profiling in microdissected hepatocellular carcinoma tissue using ProteinChip® technology. *Int J Oncol* **24:** (4) 885.

Missotten GS, Beijnen JH, Keunen JEE, Bonfrer JMG. 2003. Proteomics in uveal melanoma. *Melanoma Res* **13:** (6) 627.

Moser RJ, Reverter A, Kerr CA, Beh KJ, Lehnert SA. 2004. A mixed-model approach for the analysis of cDNA microarray gene expression data from extreme-performing pigs after infection with *Actinobacillus pleuropneumoniae*. *J Anim Sci* **82:** (5) 1261.

Muller DJ, Shinkai T, DeLuca V, Kennedy JL. 2004. Clinical implications of pharmacogenomics for tardive dyskinesia. *Pharmacogenomics J* **4:** (2) 77.

Nagahata T, Onda M, Emi M, Nagai H, Tsumagari K, Fujimoto T, Hirano A, Sato T, Nishikawa K, Akiyama F *et al*. 2004. Expression profiling to predict postoperative prognosis for estrogen receptor-negative breast cancers by analysis of 25,344 genes on a cDNA microarray. *Cancer Sci* **95:** (3) 218.

Nakamura T, Furukawa Y, Nakagawa H, Tsunoda T, Ohigashi H, Murata K, Ishikawa O, Ohgaki K, Kashimura N, Miyamoto M *et al*. 2004. Genome-wide cDNA microarray analysis of gene expression profiles in pancreatic cancers using populations of tumor cells and normal ductal epithelial cells selected for purity by laser microdissection. *Oncogene* **23:** (13) 2385.

Nakatani N, Aburatani H, Nishimura K, Semba J, Yoshikawa T. 2004. Comprehensive expression analysis of a rat depression model. *Pharmacogenomics J* **4:** (2) 114.

Navakauskiene R, Treigyte G, Gineitis A, Magnusson KE. 2004. Identification of apoptotic tyrosine-phosphorylated proteins after etoposide or retinoic acid treatment of HL-60 cells. *Proteomics* **4:** (4) 1029.

Neo S, Leow C, Vega V, Long P, Islam A, Lai P, Liu E, Ren E. 2004. Identification of discriminators of hepatoma by gene expression profiling using a minimal dataset approach. *Hepatology* **39:** (4) 944.

Newton RK, Aardema M, Aubrecht J. 2004. The utility of DNA microarrays for characterizing genotoxicity. *Environ Health Perspect* **112:** (4) 420.

Nomura F, Tomonaga T, Sogawa K, Ohashi T, Nezu M, Sunaga M, Kondo N, Iyo M, Shimada H, Ochiai T. 2004. Identification of novel and downregulated biomarkers for alcoholism by surface enhanced laser desorption/ionization-mass spectrometry. *Proteomics* **4:** (4) 1187.

Pieper R, Gatlin CL, McGrath AM, Makusky AJ, Mondal M, Seonarain M, Field E, Schatz CR, Estock MA, Ahmed N *et al*. 2004. Characterization of the human urinary proteome: A method for high-resolution display of urinary proteins on two-dimensional electrophoresis gels with a yield of nearly 1400 distinct protein spots. *Proteomics* **4:** (4) 1159.

Pierson J, Norris JL, Aerni HR, Svenningsson P, Caprioli RM, Andren PE. 2004. Molecular profiling of experimental Parkinson's disease: Direct analysis of peptides and proteins on brain tissue sections by MALDI mass spectrometry. *J Proteome Res* **3:** (2) 289.

Pusztai L, Gregory BW, Baggerly KA, Peng B, Koomen J, Kuerer HM, Esteva FJ, Symmans WF, Wagner P, Hortobagyi GN *et al*. 2004. Pharmacoproteomic analysis of prechemotherapy and postchemotherapy plasma samples from patients receiving neoadjuvant or adjuvant chemotherapy for breast carcinoma. *Cancer* **100:** (9) 1814.

Qiu J, Madoz-Gurpide J, Misek DE, Kuick R, Brenner DE, Michailidis G, Haab BB, Omenn GS, Hanash S. 2004. Development of natural

Copyright © 2004 John Wiley & Sons, Ltd. 

protein microarrays for diagnosing cancer based on an antibody response to tumor antigens. *J Proteome Res* **3:** (2) 261.
Rapp S, Baader M, Hu M, Jennen-Steinmetz C, Henn FA, Thome J. 2004. Differential regulation of synaptic vesicle proteins by antidepressant drugs. *Pharmacogenomics J* **4:** (2) 110.
Rihl M, Baeten D, Seta N, Gu J, De Keyser F, Veys EM, Kuipers JG, Zeidler H, Yu DTY. 2004. Technical validation of cDNA based microarray as screening technique to identify candidate genes in synovial tissue biopsy specimens from patients with spondyloarthropathy. *Ann Rheum Dis* **63:** (5) 498.
Sanoudou D, Kang PB, Haslett JN, Han M, Kunkel LM, Beggs AH. 2004. Transcriptional profile of postmortem skeletal muscle. *Physiol Genomics* **16:** (2) 222.
Sehata S, Kiyosawa N, Sakuma K, Ito K, Yamoto T, Teranishi M, Uetsuka K, Nakayama H, Doi K. 2004. Gene expression profiles in pregnant rats treated with T-2 toxin. *Exp Toxicol Pathol* **55:** (5) 357.
Smolenski A, Schultess J, Danielewski O, Arguinzonis MIG, Thalheimer P, Kneitz S, Walter U, Lohmann SM. 2004. Quantitative analysis of the cardiac fibroblast transcriptome-implications for NO/cGMP signaling. *Genomics* **83:** (4) 577.
Srisomsap C, Sawangareetrakul P, Subhasitanont P, Panichakul T, Keeratichamroen S, Lirdprapamongkol K, Chokchaichamnankit D, Sirisinha S, Svasti J. 2004. Proteomic analysis of cholangiocarcinoma cell line. *Proteomics* **4:** (4) 1135.
Tang K, Oeth P, Kammerer S, Denissenko MF, Ekblom J, Jurinke C, Van den Boom D, Braun A, Cantor CR. 2004. Mining disease susceptibility genes through SNP analyses and expression profiling using MALDI-TOF mass spectrometry. *J Proteome Res* **3:** (2) 218.
Thompson KL, Afshari CA, Amin RA, Bertram TA, Car B, Cunningham M, Kind C, Kramer JA, Lawton M, Mirsky M *et al*. 2004. Identification of platform-independent gene expression markers of cisplatin nephrotoxicity. *Environ Health Perspect* **112:** (4) 488.
Tsukasaki K, Tanosaki S, DeVos S, Hofmann WK, Wachsman W, Gombart AF, Krebs J, Jauch A, Bartram CR, Nagai K *et al*. 2004. Identifying progression-associated genes in adult T-cell leukemia/lymphoma by using oligonucleotide microarrays. *Int J Cancer* **109:** (6) 875.
Urs S, Smith C, Campbell B, Saxton AM, Taylor J, Zhang B, Snoddy J, Voy BJ, Moustaid-Moussa N. 2004. Gene expression profiling in human preadipocytes and adipocytes by microarray analysis. *J Nutr* **134:** (4) 762.
Valk PJM, Verhaak RGW, Beijen MA, Erpelinck CAJ, Van Doorn-Khosrovani SBV, Boer JM, Beverloo HB, Moorhouse MJ, Van der Spek PJ, Lowenberg B *et al*. 2004. Prognostically useful gene-expression profiles in acute myeloid leukemia. *N Engl J Med* **350:** (16) 1617.
Van den Eynden GG, Van der Auwera I, Van Laere S, Colpaert CG, Van Dam P, Merajver S, Kleer CG, Harris AL, Van Marck EA, Dirix LY, et al. 2004. Validation of a tissue microarray to study differential protein expression in inflammatory and non-inflammatory breast cancer. *Breast Cancer Res Treat* **85:** (1) 13.
Vitorino R, Lobo MJC, Ferrer-Correira AJ, Dubin JR, Tomer KB, Domingues PM, Amado FML. 2004. Identification of human whole saliva protein components using proteomics. *Proteomics* **4:** (4) 1109.
Waring JF, Ulrich RG, Flint N, Morfitt D, Kalkuhl A, Staedtler F, Lawton M, Beekman JM, Suter L. 2004. Interlaboratory evaluation of rat hepatic gene expression changes induced by methapyrilene. *Environ Health Perspect* **112:** (4) 439.
Xu G, Zhang WH, Bertram P, Zheng XF, McLeod H. 2004. Pharmacogenomic profiling of the P13K/PTEN-AKT-mTOR pathway in common human tumors. *Int J Oncol* **24:** (4) 893.
Young TL, Scavello GS, Paluru PC, Choi JD, Rappaport EF, Rada JA. 2004. Microarray analysis of gene expression in human donor sclera. *Mol Vis* **10:** (22-23) 163.
Zhen XA, Luke BT, Izmirlian G, Umar A, Lynch PM, Phillips RKS, Patterson S, Conrads TP, Veenstra TD, Greenwald P *et al*. 2004. Serum proteomic profiles suggest celecoxib-modulated targets and response predictors. *Cancer Res* **64:** (8) 2904.
Zhong L, Peng XJ, Hidalgo GE, Doherty DE, Stromberg AJ, Hirschowitz EA. 2004. Identification of circulating antibodies to tumor-associated proteins for combined use as markers of non-small cell lung cancer. *Proteomics* **4:** (4) 1216.

### 8 EST, cDNA and other clone resources

Aerts J, Crooijmans R, Cornelissen S, Hemmatian K, Veenendaal T, Jaadar A, Van der Poel J, Fillon V, Vignal A, Groenen M. 2004. Integration of chicken genomic resources to enable whole-genome sequencing. *Cytogenet Genome Res* **102:** (1-4) 297.
Ando K, Yamakawa S, Miyashita K, Yoshida K, Yokota A, Shinmyo A, Kohchi T. 2004. Efficient construction of cDNA microarrays utilizing normalized cDNA libraries of *Arabidopsis thaliana*. *J Biosci Bioeng* **97:** (1) 85.
Osoegawa K, Zhu BL, Shu CL, Ren T, Cao Q, Vessere GM, Lutz MM, Jensen-Seaman MI, Zhao SY, De Jong PJ. 2004. BAC resources for the rat genome project. *Genome Res* **14:** (4) 780.
Tian AG, Wang J, Cui P, Han YJ, Xu H, Cong LJ, Huang XG, Wang XL, Jiao YZ, Wang BJ *et al*. 2004. Characterization of soybean genomic features by analysis of its expressed sequence tags. *Theor Appl Genet* **108:** (5) 903.

### 9 Functional genomics

Bhaduri A, Ravishankar R, Sowdhamini R. 2004. Conserved spatially interacting motifs of protein superfamilies: Application to fold recognition and function annotation of genome data. *Proteins* **54:** (4) 657.
Kim DH, Longo M, Han Y, Lundberg P, Cantin E, Rossi JJ. 2004. Interferon induction by siRNAs and ssRNAs synthesized by phage polymerase. *Nat Biotechnol* **22:** (3) 321.
Matsuda E, Shigeoka T, Iida R, Yamanaka S, Kawaichi M, Ishida Y. 2004. Expression profiling with arrays of randomly disrupted genes in mouse embryonic stem cell leads to *in vivo* functional analysis. *Proc Natl Acad Sci U S A* **101:** (12) 4170.
Tsipouri V, Curtin JA, Nolan PM, Vizor L, Parsons CA, Clapham CM, Latham ID, Rooke LJ, Martin JE, Peters J, Hunter AJ, Rogers D, Rastan S, Brown SDM, Fisher EMC, Spurr NK, Gray IC. 2004. Three novel pigmentation mutants generated by genome-wide random ENU mutagenesis in the mouse. *Comp Funct Genom* **5:** (2) 123.

### I0 Transcriptomics

Banerjee D, Pillai B, Karnani N, Mukhopadhyay G, Prasad R. 2004. Genome-wide expression profile of steroid response in *Saccharomyces cerevisiae*. *Biochem Biophys Res Commun* **317:** (2) 406.
Boyce JD, Wilkie I, Harper M, Paustian ML, Kapur V, Adler B. 2004. Genomic-scale analysis of *Pasteurella multocida* gene expression during growth within liver tissue of chickens with fowl cholera. *Microbes Infect* **6:** (3) 290.
Ellis PJI, Furlong RA, Wilson A, Morris S, Carter D, Oliver G, Print C, Burgoyne PS, Loveland KL, Affara NA. 2004. Modulation of the mouse testis transcriptome during postnatal development and in selected models of male infertility. *Mol Hum Reprod* **10:** (4) 271.
Hayashizaki Y, Kanamori M. 2004. Dynamic transcriptome of mice. *Trends Biotechnol* **22:** (4) 161.
Jenny MJ, Ringwood AH, Schey K, Warr GW, Chapman RW. 2004. Diversity of metallothioneins in the American oyster, *Crassostrea virginica*, revealed by transcriptomic and proteomic approaches. *Eur J Biochem* **271:** (9) 1702.
Kramer JA, Curtiss SW, Kolaja KL, Alden CL, Blomme EAG, Curtiss WC, Davila JC, Jackson CJ, Bunch RT. 2004. Acute molecular markers of rodent hepatic carcinogenesis identified by transcription profiling. *Chem Res Toxicol* **17:** (4) 463.
Ma HM, Schulze S, Lee S, Yang M, Mirkov E, Irvine J, Moore P, Paterson A. 2004. An EST survey of the sugarcane transcriptome. *Theor Appl Genet* **108:** (5) 851.
Madsen SA, Chang LC, Hickey MC, Rosa GJM, Coussens PM, Burton JL. 2004. Microarray analysis of gene expression in blood neutrophils of parturient cows. *Physiol Genomics* **16:** (2) 212.
Odani M, Komatsu Y, Oka S, Iwahashi H. 2003. Screening of genes that respond to cryopreservation stress using yeast DNA microarray. *Cryobiology* **47:** (2) 155.
Roth M, Feng L, McConnell K, Schaffer P, Guerra C, Affourtit J, Piper K, Guccione L, Hariharan J, Ford M *et al*. 2004. Expression profiling

Copyright © 2004 John Wiley & Sons, Ltd. 

using a hexamer-based universal microarray. *Nat Biotechnol* **22:** (4) 418.
Su AI, Wiltshire T, Batalov S, Lapp H, Ching KA, Block D, Zhang J, Soden R, Hayakawa M, Kreiman G *et al*. 2004. A gene atlas of the mouse and human protein-encoding transcriptomes. *Proc Natl Acad Sci U S A* **101:** (16) 6062.
Walker J, Su A, Self D, Hogenesch J, Lapp H, Maier R, Hoyer D, Bilbe G. 2004. Applications of a rat multiple tissue gene expression data set. *Genome Res* **14:** (4) 742.
Wang ZQ, Dooley TP, Curto EV, Davis RL, VandeBerg JL. 2004. Cross-species application of cDNA microarrays to profile gene expression using UV-induced melanoma in *Monodelphis domestica* as the model system. *Genomics* **83:** (4) 588.
Wright JM, Zeitlin PL, Cebotaru L, Guggino SE, Guggino WB. 2004. Gene expression profile analysis of 4-phenylbutyrate treatment of IB3-1 bronchial epithelial cell line demonstrates a major influence on heat-shock proteins. *Physiol Genomics* **16:** (2) 204.
Yang JM, Kamdem DP, Keathley DE, Han KH. 2004. Seasonal changes in gene expression at the sapwood-heartwood transition zone of black locust (*Robinia pseudoacacia*) revealed by cDNA microarray analysis. *Tree Physiol* **24:** (4) 461.
Zentgraf U, Jobst J, Kolb D, Rentsch D. 2004. Senescence-related gene expression profiles of rosette leaves of *Arabidopsis thaliana*: Leaf age *versus* plant age. *Plant Biol* **6:** (2) 178.

### I1 Proteomics

Batista CVF, Del Pozo L, Zamudio FZ, Contreras S, Becerril B, Wanke E, Possani LD. 2004. Proteomics of the venom from the Amazonian scorpion *Tityus cambridgei* and the role of prolines on mass spectrometry analysis of toxins. *J Chromatogr B* **803:** (1) 55.
Blonder J, Rodriguez-Galan MC, Lucas DA, Young HA, Issaq HJ, Veenstra TD, Conrads TP. 2004. Proteomic investigation of natural killer cell microsomes using gas-phase fractionation by mass spectrometry. *Biochim Biophys Acta* **1698:** (1) 87.
Burgess SC. 2004. Proteomics in the chicken: Tools for understanding immune responses to avian diseases. *Poult Sci* **83:** (4) 552.
Chalmers MJ, Hakansson K, Johnson R, Smith R, Shen JW, Emmett MR, Marshall AG. 2004. Protein kinase A phosphorylation characterized by tandem Fourier transform ion cyclotron resonance mass spectrometry. *Proteomics* **4:** (4) 970.
Ebanks RO, Dacanay A, Goguen M, Pinto DM, Ross NW. 2004. Differential proteomic analysis of *Aeromonas salmonicida* outer membrane proteins in response to low iron and *in vivo* growth conditions. *Proteomics* **4:** (4) 1074.
Fried M, Wendler JN, Mutabingwa TK, Duffy PE. 2004. Mass spectrometric analysis of *Plasmodium falciparum* erythrocyte membrane protein-1 variants expressed by placental malaria parasites. *Proteomics* **4:** (4) 1086.
Hoa LTP, Nomura M, Kajiwara H, Day DA, Tajima S. 2004. Proteomic analysis of symbiotic differentiation of mitochondria in soybean nodules. *Plant Cell Physiol* **45:** (3) 300.
Kumar JK, Tabor S, Richardson CC. 2004. Proteomic analysis of thioredoxin-targeted proteins in *Escherichia coli*. *Proc Natl Acad Sci U S A* **101:** (11) 3759.
Lanigan MD, Vaughan JA, Shiell BJ, Beddome GJ, Michalski WP. 2004. Mycobacterial proteome extraction: Comparison of disruption methods. *Proteomics* **4:** (4) 1094.
Oh JE, Fountoulakis M, Juranville JF, Rosner M, Hengstschlaeger M, Lubec G. 2004. Proteomic determination of metabolic enzymes of the amnion cell: Basis for a possible diagnostic tool? *Proteomics* **4:** (4) 1145.
Paba J, Santana JM, Teixeira ARL, Fontes W, Sousa MV, Ricart CAO. 2004. Proteomic analysis of the human pathogen *Trypanosoma cruzi*. *Proteomics* **4:** (4) 1052.
Paron I, D'Elia A, D'Ambrosio C, Scaloni A, D'Aurizio F, Prescott A, Damante G, Tell G. 2004. A proteomic approach to identify early molecular targets of oxidative stress in human epithelial lens cells. *Biochem J* **378:** (3) 929.
Pemberton AD, Knight PA, Wright SH, Miller HRP. 2004. Proteomic analysis of mouse jejunal epithelium and its response to infection with the intestinal nematode, *Trichinelia spiralis*. *Proteomics* **4:** (4) 1101.
Richly E, Leister D. 2004. An improved prediction of chloroplast proteins reveals diversities and commonalities in the chloroplast proteomes of *Arabidopsis* and rice. *Gene* **32:** (9) 11.
Rosen R, Sacher A, Shechter N, Becher D, Buttner K, Biran D, Hecker M, Ron EZ. 2004. Two-dimensional reference map of *Agrobacterium tumefaciens* proteins. *Proteomics* **4:** (4) 1061.
Taylor CM, Marta CB, Claycomb RJ, Han DK, Rasband MN, Coetzee T, Pfeiffer SE. 2004. Proteomic mapping provides powerful insights into functional myelin biology. *Proc Natl Acad Sci U S A* **101:** (13) 4643.
Yook SH, Oltvai ZN, Barabasi AL. 2004. Functional and topological characterization of protein interaction networks. *Proteomics* **4:** (4) 928.
Zhao YX, Zhang W, Kho YJ, Zhao YM. 2004. Proteomic analysis of integral plasma membrane proteins. *Anal Chem* **76:** (7) 1817.

### I3 Metabolomics

Cakir T, Kirdar B, Ulgen KO. 2004. Metabolic pathway analysis of yeast strengthens the bridge between transcriptomics and metabolic networks. *Biotechnol Bioeng* **86:** (3) 251.
Ma HW, Zeng AP. 2004. Phylogenetic comparison of metabolic capacities of organisms at genome level. *Mol Phylogenet Evol* **31:** (1) 204.
Rasko DA, Ravel J, Okstad OA, Helgason E, Cer RZ, Jiang LX, Shores KA, Fouts DE, Tourasse NJ, Angiuoli SV *et al*. 2004. The genome sequence of *Bacillus cereus* ATCC 10987 reveals metabolic adaptations and a large plasmid related to *Bacillus anthracis* pXO1. *Nucleic Acids Res* **32:** (3) 977.
Siebers B, Tjaden B, Michalke K, Dorr C, Ahmed H, Zaparty M, Gordon P, Sensen CW, Zibat A, Klenk HP *et al*. 2004. Reconstruction of the central carbohydrate metabolism of *Thermoproteus tenax* by use of genomic and biochemical data. *J Bacteriol* **186:** (7) 2179.
Tollet-Egnell P, Parini P, Stahlberg N, Lonnstedt I, Lee NH, Rudling M, Flores-Morales A, Norstedt G. 2004. Growth hormone-mediated alteration of fuel metabolism in the aged rat as determined from transcript profiles. *Physiol Genomics* **16:** (2) 261.
Wong MS, Raab RM, Rigoutsos I, Stephanopoulos GN, Kelleher JK. 2004. Metabolic and transcriptional patterns accompanying glutamine depletion and repletion in mouse hepatoma cells: A model for physiological regulatory networks. *Physiol Genomics* **16:** (2) 247.

### I4 Genomic approaches to development

Tamborindeguy C, Ben C, Liboz T, Gentzbittel L. 2004. Sequence evaluation of four specific cDNA libraries for developmental genomics of sunflower. *Mol Genet Genomics* **271:** (3) 367.
Verhoeckx KCM, Bijlsma S, De Groene EM, Witkamp RF, Van der Greef J, Rodenburg RJT. 2004. A combination of proteomics, principal component analysis and transcriptomics is a powerful tool for the identification of biomarkers for macrophage maturation in the U937 cell line. *Proteomics* **4:** (4) 1014.
Welsh GI, Griffiths MR, Webster KJ, Page MJ, Tavare JM. 2004. Proteome analysis of adipogenesis. *Proteomics* **4:** (4) 1042.
Wesolowski SR, Raney NE, Ernst CW. 2004. Developmental changes in the fetal pig transcriptome. *Physiol Genomics* **16:** (2) 268.

### I5 Technological advances

Allmer J, Markert C, Stauber EJ, Hippler M. 2004. A new approach that allows identification of intron-split peptides from mass spectrometric data in genomic databases. *FEBS Lett* **562:** (1-3) 202.
Anderson NL, Haines LR, Pearson TW. 2004. An effective and rapid method for functional characterization of immunoadsorbents using POROS beads and flow cytometry. *J Proteome Res* **3:** (2) 228.
Anderson NL, Anderson NG, Haines LR, Hardie DB, Olafson RW, Pearson TW. 2004. Mass spectrometric quantitation of peptides and proteins using stable isotope standards and capture by anti-peptide antibodies (SISCAPA). *J Proteome Res* **3:** (2) 235.
Baczek T. 2004. Fractionation of peptides in proteomics with the use of pI-based approach and ZipTip pipette tips. *J Pharmaceut Biomed Anal* **34:** (5) 851.

Copyright © 2004 John Wiley & Sons, Ltd. 

Beer I, Barnea E, Ziv T, Admon A. 2004. Improving large-scale proteomics by clustering of mass spectrometry data. *Proteomics* **4:** (4) 950.

Burke PV, Lai LC, Kwast KE. 2004. A rapid filtration apparatus for harvesting cells under controlled conditions for use in genome-wide temporal profiling studies. *Anal Biochem* **328:** (1) 29.

Fedrigo O, Naylor G. 2004. A gene-specific DNA sequencing chip for exploring molecular evolutionary change. *Nucleic Acids Res* **32:** (3) 1208.

Gao XL, Gulari E, Zhou XC. 2004. *In situ* synthesis of oligonucleotide microarrays. *Biopolymers* **73:** (5) 579.

Gevaert K, Ghesquiere B, Staes A, Martens L, Van Damme J, Thomas GR, Vandekerckhove J. 2004. Reversible labeling of cysteine-containing peptides allows their specific chromatographic isolation for non-gel proteome studies. *Proteomics* **4:** (4) 897.

Goodsaid FM, Smith RJ, Rosenblum IY. 2004. Quantitative PCR deconstruction of discrepancies between results reported by different hybridization platforms. *Environ Health Perspect* **112:** (4) 456.

Jones DB, Hutchinson MH, Middelberg APJ. 2004. Screening protein refolding using surface plasmon resonance. *Proteomics* **4:** (4) 1007.

Kato M, Sakai-Kato K, Jin HM, Kubota K, Miyano H, Toyo'oka T, Dulay MT, Zare RN. 2004. Integration of on-line protein digestion, peptide separation, and protein identification using pepsin-coated photopolymerized sol-gel columns and capillary electrophoresis/mass spectrometry. *Anal Chem* **76:** (7) 1896.

Kenzelmann M, Klaren R, Hergenhahn M, Bonrouhi M, Grone HJ, Schmid W, Schutz G. 2004. High-accuracy amplification of nanogram total RNA amounts for gene profiling. *Genomics* **83:** (4) 550.

Lee H, Yi EC, Wen B, Reily TP, Pohl L, Nelson S, Aebersold R, Goodlett DR. 2004. Optimization of reversed-phase microcapillary liquid chromatography for quantitative proteomics. *J Chromatogr B* **803:** (1) 101.

Li LH, Li JC, Lin YF, Lin CY, Chen CY, Tsai SF. 2004. Genomic shotgun array: A procedure linking large-scale DNA sequencing with regional transcript mapping - Online art. no. e27. *Nucleic Acids Res* **32:** (3) e27.

Liu RH, Yang JN, Lenigk R, Bonanno J, Grodzinski P. 2004. Self-contained, fully integrated biochip for sample preparation, polymerase chain reaction amplification, and DNA microarray detection. *Anal Chem* **76:** (7) 1824.

Mah N, Thelin A, Lu T, Nikolaus S, Kuhbacher T, Gurbuz Y, Eickhoff H, Kloppel G, Lehrach H, Mellgard B *et al*. 2004. A comparison of oligonucleotide and cDNA-based microarray systems. *Physiol Genomics* **16:** (3) 361.

Mattes WB. 2004. Annotation and cross-indexing of array elements on multiple platforms. *Environ Health Perspect* **112:** (4) 506.

Park CH, Jeong HJ, Jung JJ, Lee GY, Kim SC, Kim TS, Yang SH, Chung HC, Rha SY. 2004. Fabrication of high quality cDNA microarray using a small amount of cDNA. *Int J Mol Med* **13:** (5) 675.

Pinhasov A, Mei J, Amaratunga D, Amato FA, Lu H, Kauffman J, Xin H, Brenneman DE, Johnson DL, Andrade-Gordon P *et al*. 2004. Gene expression analysis for high throughput screening applications. *Comb Chem High Throughput Scr* **7:** (2) 133.

Redman JC, Haas BJ, Tanimoto G, Town CD. 2004. Development and evaluation of an *Arabidopsis* whole genome Affymetrix probe array. *Plant J* **38:** (3) 545.

Richert S, Luche S, Chevallet M, Van Dorsselaer A, Leize-Wagner E, Rabilloud T. 2004. About the mechanism of interference of silver staining with peptide mass spectrometry. *Proteomics* **4:** (4) 909.

Rockett JC, Hellmann GM. 2004. Confirming microarray data - Is it really necessary? *Genomics* **83:** (4) 541.

Rodriguez-Pineiro AM, Ayude D, Rodriguez-Berrocal FJ, De la Cadena MP. 2004. Concanavalin A chromatography coupled to two-dimensional gel electrophoresis improves protein expression studies of the serum proteome. *J Chromatogr B* **803:** (2) 337.

Rosenzweig BA, Pine PS, Domon OE, Morris SM, Chen JJ, Sistare FD. 2004. Dye-bias correction in dual-labeled cDNA microarray gene expression measurements. *Environ Health Perspect* **112:** (4) 480.

Scherl A, Francois P, Converset V, Bento M, Burgess JA, Sanchez JC, Hochstrasser DF, Schrenzel J, Corthals GL. 2004. Nonredundant mass spectrometry: A strategy to integrate mass spectrometry acquisition and analysis. *Proteomics* **4:** (4) 917.

Skilling J, Denny R, Richardson K, Young P, McKenna T, Campuzano I, Ritchie M. 2004. ProbSeq - A fragmentation model for interpretation of electrospray tandem mass spectrometry data. *Comp Funct Genom* **5:** (1) 61.

Smirnov DA, Burdick JT, Morley M, Cheung VG. 2004. Method for manufacturing whole-genome microarrays by rolling circle amplification. *Gene Chromosomes Cancer* **40:** (1) 72.

Tannu NS, Wu J, Rao VK, Gadgil HS, Pabst MJ, Gerling IC, Raghow R. 2004. Paraffin-wax-coated plates as matrix-assisted laser desorption/ionization sample support for high-throughput identification of proteins by peptide mass fingerprinting. *Anal Biochem* **327:** (2) 222.

Vainrub A, Pettitt BM. 2004. Theoretical aspects of genomic variation screening using DNA microarrays. *Biopolymers* **73:** (5) 614.

Van Eijk R, Oosting J, Sieben N, Van Wezel T, Cleton-Jansen AM. 2004. Visualization of regional gene expression biases by microarray data sorting. *Biotechniques* **36:** (4) 592.

### I6 Bioinformatics

Adindla S, Inampudi KK, Guruprasad K, Guruprasad L. 2004. Identification and analysis of novel tandem repeats in the cell surface proteins of archaeal and bacterial genomes using computational tools. *Comp Funct Genom* **5:** (1) 2.

Antonov AV, Tetko IV, Mader MT, Budczies J, Mewes HW. 2004. Optimization models for cancer classification: Extracting gene interaction information from microarray expression data. *Bioinformatics* **20:** (5) 644.

Baggerly KA, Morris JS, Coombes KR. 2004. Reproducibility of SELDI-TOF protein patterns in serum: Comparing datasets from different experiments. *Bioinformatics* **20:** (5) 777.

Barash Y, Dehan E, Krupsky M, Franklin M, Geraci M, Friedman N, Kaminski N. 2004. Comparative analysis of algorithms for signal quantitation from oligonucleotide microarrays. *Bioinformatics* **20:** (6) 839.

Bikandi J, San Millan R, Rementeria A, Garaizar J. 2004. *In silico* analysis of complete bacterial genomes: PCR, AFLP-PCR and endonuclease restriction. *Bioinformatics* **20:** (5) 798.

Blanchette M, Kent WJ, Riemer C, Elnitski L, Smit AFA, Roskin KM, Baertsch R, Rosenbloom K, Clawson H, Green ED *et al*. 2004. Aligning multiple genomic sequences with the threaded blockset aligner. *Genome Res* **14:** (4) 708.

Bo TH, Dysvik J, Jonassen I. 2004. LSimpute: Accurate estimation of missing values in microarray data with least squares methods - Online art. no. e34. *Nucleic Acids Res* **32:** (3) e34.

Bray N, Pachter L. 2004. MAVID: Constrained ancestral alignment of multiple sequences. *Genome Res* **14:** (4) 693.

Chakrabarti K, Pachter L. 2004. Visualization of multiple genome annotations and alignments with the K-BROWSER. *Genome Res* **14:** (4) 716.

Chen D, Lin S, Soong S. 2004. Gene selection for oligonucleotide array: An approach using PM probe level data. *Bioinformatics* **20:** (6) 854.

Choi C, Krull M, Kel A, Kel-Margoulis O, Pistor S, Potapov A, Voss N, Wingender E. 2004. TRANSPATH® - A high quality database focused on signal transduction (Conference Paper). *Comp Funct Genom* **5:** (2) 163.

De Las Rivas J, De Luis A. 2004. Interactome data and databases: Different types of protein interaction (Conference Paper). *Comp Funct Genom* **5:** (2) 173.

Deng MH, Tu ZD, Sun FZ, Chen T. 2004. Mapping gene ontology to proteins based on protein-protein interaction data. *Bioinformatics* **20:** (6) 895.

Dugas M, Merk S, Breit S, Dirschedl P. 2004. mdclust - Exploratory microarray analysis by multidimensional clustering. *Bioinformatics* **20:** (6) 931.

Eilers PHC, Goeman JJ. 2004. Enhancing scatterplots with smoothed densities. *Bioinformatics* **20:** (5) 623.

Frangeul L, Glaser P, Rusniok C, Buchrieser C, Duchaud E, Dehoux PE, Kunst F. 2004. CAAT-Box, contigs-Assembly and Annotation Tool-Box for genome sequencing projects. *Bioinformatics* **20:** (5) 790.

Frommlet F, Futschik A, Bogdan M. 2004. On the significance of sequence alignments when using multiple scoring matrices. *Bioinformatics* **20:** (6) 881.

Gilchrist M, Salter L, Wagner A. 2004. A statistical framework for com-

Copyright © 2004 John Wiley & Sons, Ltd. 

bining and interpreting proteomic datasets. *Bioinformatics* **20:** (5) 689.
Grover D, Mukerji M, Bhatnagar P, Kannan K, Brahmachari SK. 2004. Alu repeat analysis in the complete human genome: trends and variations with respect to genomic composition. *Bioinformatics* **20:** (6) 813.
Hall R, Stern L. 2004. A rapid method for illustrating features in both coding and non-coding regions of a genome. *Bioinformatics* **20:** (6) 982.
Havlak P, Chen R, Durbin KJ, Egan A, Ren YR, Song XZ, Weinstock GM, Gibbs RA. 2004. The atlas genome assembly system. *Genome Res* **14:** (4) 721.
Hourai Y, Akutsu T, Akiyama Y. 2004. Optimizing substitution matrices by separating score distributions. *Bioinformatics* **20:** (6) 863.
Kalafus KJ, Jackson AR, Milosavljevic A. 2004. Pash: Efficient genome-scale sequence anchoring by Positional Hashing. *Genome Res* **14:** (4) 672.
Keith JM, Cochran DAE, Lala GH, Adams P, Bryant D, Mitchelson KR. 2004. Unlocking hidden genomic sequence - Online art. no. e35. *Nucleic Acids Res* **32:** (3) e35.
Khan HA. 2004. ArraySolver: An algorithm for colour-coded graphical display and Wilcoxon signed-rank statistics for comparing microarray gene expression data. *Comp Funct Genom* **5:** (1) 39.
Kihara D, Jeffrey S. 2004. Microbial genomes have over 72% structure assignment by the threading algorithm PROSOPECTOR_Q. *Proteins* **55:** (2) 464.
Lavigne R, Sun WD, Volckaert G. 2004. PHIRE, a deterministic approach to reveal regulatory elements in bacteriophage genomes. *Bioinformatics* **20:** (5) 629.
Lavine BK, Davidson CE, Rayens WS. 2004. Machine learning based pattern recognition applied to microarray data. *Comb Chem High Throughput Scr* **7:** (2) 115.
Lee JK, Laudeman T, Kanter J, James T, Siadaty MS, Knaus WA, Prorok A, Bao YD, Freeman B, Puiu D *et al*. 2004. GeneX Va: VBC open source microarray database and analysis software. *Biotechniques* **36:** (4) 634.
Lisacek F, Chichester C, Gonnet P, Jaillet O, Kappus S, Nikitin F, Roland P, Rossier G, Truong L, Appel R. 2004. Shaping biological knowledge: Applications in proteomics (Conference Papers). *Comp Funct Genom* **5:** (2) 190.
Majeed AA, Ahmed N, Rao KR, Ghousunnissa S, Kauser F, Bose B, Nagarajaram HA, Katoch VM, Cousins DV, Sechi LA *et al*. 2004. AmpliBASE MT™: A *Mycobacterium tuberculosis* diversity knowledgebase. *Bioinformatics* **20:** (6) 989.
Martin ACR. 2004. PDBSprotEC: A web-accessible database linking PDB chains to EC numbers *via* SwissProt. *Bioinformatics* **20:** (6) 986.
Mattes WB, Pettit SD, Sansone SA, Bushel PR, Waters MD. 2004. Database development in toxicogenomics: Issues and efforts. *Environ Health Perspect* **112:** (4) 495.
Medjahed D, Lemkin PF, Smythers GW, Munroe DJ. 2004. Looking for cancer clues in publicly accessible databases (Conference Paper). *Comp Funct Genom* **5:** (2) 196.
Nasi S. 2004. From databases to modelling of functional pathways (Conference Paper). *Comp Funct Genom* **5:** (2) 179.
Nilsson J, Fioretos T, Hoglund M, Fontes M. 2004. Approximate geodesic distances reveal biologically relevant structures in microarray data. *Bioinformatics* **20:** (6) 874.
Ouyang M, Welsh WJ, Georgopoulos P. 2004. Gaussian mixture clustering and imputation of microarray data. *Bioinformatics* **20:** (6) 917.
Paul N, Kellenberger E, Bret G, Muller P, Rognan D. 2004. Recovering the true targets of specific ligands by virtual screening of the Protein Data Bank. *Proteins* **54:** (4) 671.
Price MN, Rieffel E. 2004. Finding coexpressed genes in counts-based data: An improved measure with validation experiments. *Bioinformatics* **20:** (6) 945.
Razumovskaya J, Olman V, Xu D, Uberbacher EC, VerBerkmoes NC, Hettich RL, Xu Y. 2004. A computational method for assessing peptide-identification reliability in tandem mass spectrometry analysis with SEQUEST. *Proteomics* **4:** (4) 961.
Rhodes DR, Yu JJ, Shanker K, Deshpande N, Varambally R, Ghosh D, Barrette T, Pandey A, Chinnaiyan AM. 2004. ONCOMINE: A cancer microarray database and integrated data-mining platform. *Neoplasia* **6:** (1) 1.
Robinson PN, Wollstein A, Bohme U, Beattie B. 2004. Ontologizing gene-expression microarray data: Characterizing clusters with Gene Ontology. *Bioinformatics* **20:** (6) 979.
Romano P, Aresu O, Manniello MA, Parodi B. 2004. Interoperability of CABRI services and biochemical pathways databases (Conference Paper). *Comp Funct Genom* **5:** (2) 169.
Romero PR, Karp PD. 2004. Using functional and organizational information to improve genome-wide computational prediction of transcription units on pathway-genome databases. *Bioinformatics* **20:** (5) 709.
Roth V, Lange T. 2004. Bayesian class discovery in microarray datasets. *IEEE Trans Biomed Eng* **51:** (5) 707.
Sadreyev RI, Grishin NV. 2004. Quality of alignment comparison by COMPASS improves with inclusion of diverse confident homologs. *Bioinformatics* **20:** (6) 818.
Sakharkar KR, Chow VTK. 2004. Exploring genome architecture through GOV: A WWW-based gene order visualizer. *Bioinformatics* **20:** (6) 984.
Schoof H, Ernst R, Mayer KFX. 2004. The PlaNet consortium: A network of European plant databases connecting plant genome data in an integrated biological knowledge resource (Conference Paper). *Comp Funct Genom* **5:** (2) 184.
Shah N, Couronne O, Pennacchio LA, Brudno M, Batzoglou S, Bethel EW, Rubin EM, Hamann B, Dubchak I. 2004. Phylo-VISTA: interactive visualization of multiple DNA sequence alignments. *Bioinformatics* **20:** (5) 636.
Shmulevich I, Gluhovsky I, Hashimoto RF, Dougherty ER, Zhang W. 2003. Steady-state analysis of genetic regulatory network modelled by probabilistic Boolean networks. *Comp Funct Genom* **4:** (6) 601.
Smoot ME, Guerlain SA, Pearson WR. 2004. Visualization of near-optimal sequence alignments. *Bioinformatics* **20:** (6) 953.
Stein WD, Litman T, Fojo T, Bates SE. 2004. Serial Analysis of Gene Expression (SAGE) database analysis of chemosensitivity: Comparing solid tumors with cell lines and comparing solid tumors from different tissue origins. *Cancer Res* **64:** (8) 2805.
Sun H, Davuluri RV. 2004. Java-based application framework for visualization of gene regulatory region annotations. *Bioinformatics* **20:** (5) 727.
Sutcliffe IC, Harrington DJ. 2004. Putative lipoproteins of *Streptococcus agalactiae* identified by bioinformatic genome analysis. *Antonie van Leeuwenhoek* **85:** (4) 305.
Tammi MT, Arner E, Kindlund E, Andersson B. 2004. ReDiT: Repeat Discrepancy Tagger - a shotgun assembly finishing aid. *Bioinformatics* **20:** (5) 803.
Taneda A. 2004. Adplot: detection and visualization of repetitive patterns in complete genomes. *Bioinformatics* **20:** (5) 701.
Tang C, Zhang A, Ramanathan M. 2004. ESPD: A pattern detection model underlying gene expression profiles. *Bioinformatics* **20:** (6) 829.
Tress ML, Grana O, Valencia A. 2004. SQUARE - Determining reliable regions in sequence alignments. *Bioinformatics* **20:** (6) 974.
Van Beek JHGM. 2004. Data integration and analysis for medical systems biology (Conference Paper). *Comp Funct Genom* **5:** (2) 201.
Weiller GF, Caraux G, Sylvester N. 2004. The model distribution of protein isoelectric points reflects amino acid properties rather than sequence evolution. *Proteomics* **4:** (4) 943.
Wiederkehr C, Basavaraj R, De Menthiere CS, Koch R, Schlecht U, Hermida L, Masdoua B, Ishii R, Cassen V, Yamamoto M *et al*. 2004. Database model and specification of GermOnline Release 2.0, a cross-species community annotation knowledgebase on germ cell differentiation. *Bioinformatics* **20:** (5) 808.
Wittig U, Weidemann A, Kania R, Peiss C, Rojas I. 2004. Classification of chemical compounds to support complex queries in a pathway database (Conference Paper). *Comp Funct Genom* **5:** (2) 156.
Xie HQ, Diber A, Pollock S, Nemzer S, Safer H, Meloon B, Olson A, Hwang JJ, Endress GA, Savitsky K *et al*. 2004. Bridging expressed sequence alignments through targeted cDNA sequencing. *Genomics* **83:** (4) 572.
Yang ZR, Chou KC. 2004. Bio-support vector machines for computational proteomics. *Bioinformatics* **20:** (5) 735.
Yeung LK, Szeto LK, Liew AWC, Yan H. 2004. Dominant spectral component analysis for transcriptional regulations using microarray time-series data. *Bioinformatics* **20:** (5) 742.
Zhang R, Zhang CT. 2004. A systematic method to identify genomic islands and its applications in analyzing the genomes of *Corynebacterium glutamicum* and *Vibrio vulnificus* CMCP6 chromosome I. *Bioinformatics* **20:** (5) 612.

Copyright © 2004 John Wiley & Sons, Ltd. 
